# 70. Sporotrichosis of the tongue

**DOI:** 10.1093/rap/rky034.033

**Published:** 2018-09-20

**Authors:** Katie Macdonald, James Galloway

**Affiliations:** Rheumatology, King’s College Hospital, London, UNITED KINGDOM

**Introduction:** We describe the first reported case of oral and pulmonary sporotrichosis in a man prescribed anti-tumour necrosis factor therapy for psoriatic arthritis. The case highlights the importance of vigilance for opportunistic infections in immunosuppressed patients in whom unusual infections can present and in an atypical manner.

**Case description:** A 72 year old man presented with tongue ulceration and swelling. He had a past medical history of psoriatic arthritis for which he received adalimumab 40mg alternate weeks and methotrexate 17.5mg weekly. He had been treated for latent tuberculosis prior to treatment. He is retired having previously travelled extensively in his job for a non-governmental organisation; his last travel had been the year before to South Africa where he had been on safari. He was an ex-smoker and drank a moderate amount of alcohol. He described a four-month history of tongue ulceration, which initially started with midline swelling of the anterior dorsum of the tongue. Ulceration then spread to the right and ventral aspect. There was a grey and yellow slough that covered the tongue and this was the same colour as his phlegm. He had a productive cough but did not complain of breathlessness and he had no fever. Over the last few months he had weight loss of eleven kilograms, which he attributed to his difficulty to chew and swallow. Earlier in the course of the illness he had presented to a private clinic to see an oral surgeon who had performed a biopsy. The histology showed numerous hyphae in the inflamed corium, numerous granulomata some of which had caseation, and filamentous organisms on staining with periodic acid-Schiff within the corium but no organisms identified with either Gram stain or Ziehl Neelsen stain. No malignant features were seen. Amoxicillin, fluconazole and nystatin was started but without any benefit and the ulceration and swelling developed further. He was admitted under the medical take for further investigation and intravenous fluid. At this point the tongue was swollen to the extent that it almost filled the entire oral cavity. Extensive ulceration covered the anteroventral aspect and extended into the floor of the mouth. Submandibular lymphadenopathy was palpable. Cardiovascular, respiratory and abdominal examinations were normal. He had no peripheral skin lesions or rash. Laboratory tests revealed a raised C-reactive protein at 34.1 mg/L and lymphopaenia 1.23 10^9/L. Liver biochemistry and renal function were normal on admission. Haemoglobin was 134 g/L with a raised mean cell volume (110.1 fL). Serum Beta-D-Glucan was <8 pg/ml. Computed tomography of the neck, thorax, abdomen and pelvis confirmed the oral lesion and a foci of ground glass changes in the right upper lobe and left lower lobe. The lung lesions prompted a bronchoscopy and bronchial washings found numerous macrophages but staining was negative for microorganisms including pneumocystis. An incisional biopsy was repeated and found granulomatous inflammation with necrosis and hyphae and spores. Culture isolated an organism resembling pale, grey yeast and this sample was sent to the United Kingdom Mycology Reference Laboratory. The tongue isolate and broncho-alveolar washing culture both isolated *Sporothrix shenckii*. Speciation was confirmed by polymerase chain reaction in the reference laboratory. The organism was resistant to itraconazole and this was initiated with improvement seen within one week. We plan to treat for a twelve-month course and will consider longer in view of plans for ongoing immunosuppression.

**Discussion:** Sporothrix is a thermally dimorphic fungus, transitioning between hyphal and yeast forms at varying temperature. The organism is found in soil worldwide usually in it the hyphae form whereas at body temperature the yeast form predominates. The organism classically causes nodular ulcerating lesions on skin, which typically occur after an inoculation from a thorn, thus the eponymous name ‘rose gardener’s disease’. We learnt the importance of keeping a broad differential list and using clinical acumen to interpret the significance of test. For example, certain results early on in the admission, such as the negative beta D glucan and negative panfungal polymerase chain reaction test, led me to disregard fungal infections as possible cause. However, greater understanding of how these tests work would have meant applying these tests more appropriately. Beta D glucan is a component of the cell wall of fungi and detection in serum samples demonstrate disseminated infection. Despite two foci of infection the fungi had likely spread through the airways and not haematogenously and therefore was not be detectable in the blood stream.

**Key Learning Points:** This case has taught us the importance of being vigilant for unusual infections in patients on biologic therapy. Our reliance on our oral medical and microbiology colleagues for their knowledge of mycology and acumen in interpreting tests highlighted how vital a multidisciplinary approach is, particularly when investigating patients with a broad differential diagnosis.

**Disclosure: K. Macdonald:** None. **J. Galloway:** None.

